# Opioids and alpha-2-agonists for analgesia and sedation in newborn infants: protocol of a systematic review

**DOI:** 10.1186/s13643-020-01436-0

**Published:** 2020-08-20

**Authors:** Mari Kinoshita, Katarzyna Stempel, Israel Junior Borges do Nascimento, Dhashini Naidu Vejayaram, Elisabeth Norman, Matteo Bruschettini

**Affiliations:** 1 Faculty of Medicine, Lund University, Lund, Sweden; 2 Keio University School of Medicine, Tokyo, Japan; 3 School of Medicine at Universidade Federal de Minas Gerais, Minas Gerais Belo Horizonte, Brazil; 4 Medical College of Wisconsin, Milwaukee, WI USA; 5 Lund University, Skane University Hospital, Department of Clinical Sciences Lund, Pediatrics, Lund, Sweden; 6 Cochrane Sweden, Skåne University Hospital, Lund, Sweden

**Keywords:** Opioids, Alpha-2-agonists, Neonate, Sedation, Analgesia, Clonidine, Dexmedetomidine, Morphine, Fentanyl, Pharmacokinetics

## Abstract

**Background:**

Hospitalized newborn infants may require analgesia and sedation either for the management of procedural pain, during or after surgery, and other painful conditions. The benefits and harms of opioids administered at different doses and routes of administration have been reported in numerous trials and systematic reviews. The use of alpha-2-agonists such as clonidine and dexmedetomidine in newborn infants is more recent, and they might be prescribed to reduce the total amount of opioids which are thought to have more side effects. Moreover, alpha-2-agonists might play an important role in the management of agitation and discomfort.

**Methods:**

We will conduct a systematic review and meta-analysis on the use of opioids, alpha-2-agonists, or the combination of both drugs. We will include randomized controlled trials to assess benefits and harms and observational studies to assess adverse events and pharmacokinetics; preterm and term infants; studies on any opioids or alpha-2-agonists administered for any indication and by any route except spinal, intraosseous, or administration for nerve blocks and wound infusions. The use of opioids or alpha-2-agonists will be compared to no intervention; placebo with normal saline or other non-sedative, non-analgesic drug; control with oral sugar solution or non-pharmacological intervention; same drug of different dose or route; or a different drug (not limiting to opioids and alpha-2-agonists) or combinations of such drugs. The primary outcomes for this review will be all-cause mortality during initial hospitalization and hypotension requiring medical therapy. We will conduct a search in the following databases: The Cochrane Central Register of Controlled Trials (CENTRAL, The Cochrane Library), MEDLINE, Embase, and CINAHL. Two review authors will independently screen records for inclusion, undertake data abstraction using a data extraction form and assess the risk of bias of all included trials using the Cochrane “Risk of bias” tool.

**Discussion:**

This systematic review will summarize and update our knowledge about neonatal analgesia and sedation including pharmacokinetics/pharmacodynamics, and provide a platform for developing evidence-based guidelines that we can immediately apply to our clinical practice.

**Systematic review registration:**

PROSPERO 2020 CRD42020170852

## Background

### Description of the condition

Newborn infants in the neonatal intensive care unit (NICU) are not just ill but constantly exposed to various stimuli through repeated invasive procedures, physical handling, and the extrauterine environment itself for those that are born preterm. A systematic review in 2008 looking into the number of painful procedures reported that a NICU patient received a median of 10 (interquartile range (IQR) 5-17) per day during the first 2 weeks of hospitalization; a later review in 2016 reported a similar average of 7.5 to 17.3 per day, and the 8 years difference in publication did not change the fact that the procedures were often accompanied by inconsistent and inadequate analgesia [1, 2]. All these stimuli result in acute pain and stress, and also easily mount to chronic pain due to hyperalgesia during a vital period of complex brain development. The plasticity of the neonatal brain increases its vulnerability to these early adverse events, thereby leading to abnormal neurodevelopmental, behavioral, and cognitive outcomes [3–8]. Moreover, premature infants with even more immature brains are already predisposed to developing such sequelae from inadequately treated pain, while being most likely to be exposed to more pain during their longer NICU hospitalization. The unique characteristic of the neonatal population strengthens the rationale to establish a therapeutic approach for adequate analgesia and sedation.

### Description of the intervention

Various interventions to reduce pain have been studied, but clinical protocols and guidelines still differ a lot. For short, mild to moderately painful procedures, the use of non-pharmacological strategies should always be considered [9]. For moderate to severe painful conditions, opioids have traditionally been used in the NICU, although with several side effects such as respiratory depression, hypotension, constipation, and also development of tachyphylaxis and abstinence. Opioids are also often used for sedation to reduce stress during mechanical ventilation.

The opioids most often used during neonatal intensive care are morphine, fentanyl, and remifentanil. The fentanyl derivates alfentanil and sufentanil are more randomly used. The opioids have varying pharmacokinetic (PK) and pharmacodynamic (PD) profiles and should optimally be administered in an individualized way according to the need, clinical state, and expected development of the hospitalization. Fentanyl and remifentanil are administered intravenously in the very sick infants, whereas morphine can be administered by both intravenous and oral routes.

Morphine has the longest duration of onset, half-life, and elimination time, followed by fentanyl and remifentanil [10–12]. Remifentanil is a short-acting opioid with ultra-rapid onset and very fast elimination profile, very suitable for rapid painful procedures such as tracheal intubations [13]. PD studies on opioids report on hypotension as the most adverse effect [10]. Several larger studies have questioned the effect and reported on a negative outcome [14–16] and study data report on the negative impact on the structure and function of the developing brain including neuronal apoptosis [17–19].

To reduce the opioid doses, additive treatment with paracetamol and alpha-2-agonists (clonidine and dexmedetomidine) has been implemented in the NICU [20, 21]. The analgesic and sedative properties of the alpha-2-agonists without respiratory and gastrointestinal side effects, along with the potential beneficial effects to the immature brain, have promoted their use to enable reduced opioid dosage or even to replace opioid administration [21–25].

Clonidine is administered intravenously or orally, and it was initially used to treat neonatal opioid abstinence [26]. Clonidine reduces fentanyl and midazolam demand by inducing deeper levels of analgesia and sedation without substantial side effects in ventilated newborn infants [23].

Dexmedetomidine is intravenously administered. There are dexmedetomidine PK data in newborns indicating longer half-lives, lower clearance, and smaller doses required for adequate effects in infants of lower age [27, 28]. Study data on dexmedetomidine report self-limited bradycardia and hypotension which do not require discontinuation of the drug: dexmedetomidine should be considered as safe in this population [21, 27–31]. In studies of dexmedetomidine used in combination with opioids for postoperative care, opioid-sparing effects are reported [21, 29].

Electroencephalogram (EEG) has been used to evaluate sedative and analgesic drug effects to the brain (newborn brain), and show significant background depression [32] and in some cases epileptiform activity and seizures [33, 34]. In some of the studies, neurodepression is related to drug-induced negative hemodynamic changes as assessed by near-infrared spectroscopy (NIRS) [35].

Aiming at more individualized pharmacologic treatment, pharmacogenetic studies are needed. Single-nucleotide polymorphisms in genes involved in pain control might predispose to exaggerated sensitivity or difference in opioid analgesic effect [12].

### How the intervention might work

Opioids work by binding to mu, kappa, and delta-opioid receptors and modulating nociceptive information at both peripheral and central sites, thus promoting a temporary pain relief that is dose dependent and well tolerated [36]. In addition to analgesic effects, opioids demonstrate sedative effects through generalized central nervous depression though its precise mechanism remains unknown.

In contrast, dexmedetomidine and clonidine are alpha-2-agonists that have analgesic and sedative properties that work through a completely different mechanism involving the brainstem and the spinal cord. Dexmedetomidine is eight to ten times more specific for alpha-2-adrenergic receptors than clonidine [37], but both drugs act by stimulating presynaptic alpha-2-adrenoceptors which consequently decreases synaptic noradrenaline concentrations. Inhibitory signals are transmitted from locus coeruleus in the brainstem through the descending reticulospinal tracts, as well as from dorsal horn neurons, and thus reducing substance P [38]. Since opioids and alpha-2-agonists are different types of analgosedatives, the combination of both drugs can be used to reduce doses of the drug with anticipated side effects.

### Why is it important to do this review

Inadequate pain management in early human life leads to neurologic and neuropsychologic sequelae, while exposure to analgosedatives during the same period is associated with neuroapoptosis in animal experiments and affected neurodevelopment in human studies [18, 19]. Both aspects contribute to the impaired neurodevelopmental outcome; therefore, the current practice of analgosedatives remains to be reconsidered and tailored to reach effective control of neonatal pain with minimal harm to the most vulnerable patients. There is a call for a systematic review with the available evidence on opioids and alpha-2-agonists to better provide clinical guidance for neonatal pain management.

### Objectives

The aim of this systematic review is to summarize the best available evidence regarding the use of opioids, alpha-2-agonists, or the combination of both for the optimal management of pain and distress in critically ill infants in the NICU.

## Methods/design

We will conduct a systematic review using the standard methods of the Cochrane Neonatal Review Group (neonatal.cochrane.org/resourcesauthors/author-resources-new-reviews). The protocol for this review has been registered to the International Prospective Register of Systematic Reviews (PROSPERO; CRD42020170852) and will follow the reporting guideline by the Preferred Reporting Items for Systematic Review and Meta-Analyses for Protocols (PRISMA-P) (Additional file 1). There is no need for an ethical approval for this study due to the inherent design of a systematic review.

### Types of studies

We will include all randomized and quasi-randomized controlled trials (RCTs), observational studies if reporting pharmacokinetics data. Cross-over and cluster-randomized trials will be excluded.

### Types of participants

Our population of interest are preterm and term infants of a postmenstrual age (PMA) up to 46 weeks and 0 days, irrespective of their gestational age at birth. We will exclude data on infants who are already receiving sedative or analgesic drugs, infants treated for neonatal abstinence syndrome, and infants undergoing dialysis. If a study enrolls patients both within and outside of our established target population, i.e., infants up to PMA 46 weeks and 0 days and those above PMA 46 weeks and 1 day are included together in the study, we will attempt to contact the authors of said study in order to obtain full data for each age group and include only data concerning infants that meet the inclusion criteria.

### Types of interventions

We will include studies on any opioids, alpha-2-agonists (i.e., clonidine and dexmedetomidine), or the combination of both administered for any indication and by any other route than spinal (i.e., intrathecal, epidural, caudal) or intraosseous, also excluding administration for nerve blocks and wound infusions. The comparators or controls are no intervention; placebo with normal saline or other non-sedative, non-analgesic drug; control with oral sugar solution or non-pharmacological intervention (skin-to-skin contact, music exposure, non-nutritive sucking, swaddling, etc.); same drug of different dose or route; or a different drug (including drugs other than opioids and alpha-2-agonists) or combinations of such drugs.

### Types of outcome measures: primary outcomes, secondary outcomes

The primary outcomes for this review are all-cause mortality during initial hospitalization, and circulatory instability, defined as hypotension requiring medical therapy (vasopressors, fluid boluses, or any changes in such drug administration).

The secondary outcomes measured are all-cause neonatal mortality (death until postnatal day 28); episodes of bradycardia defined as a fall in heart rate of more than 30% below the baseline or less than 100 beats per minute for 10 s or longer, and respiratory depression, that is episodes of apnea; neonatal pain and sedation during the administration of selected drugs, assessed with validated scales for procedural pain (the Neonatal Facial Coding System (NFCS) [39], the Neonatal Infant Pain scale (NIPS) [40], the Premature Infant Pain Profile (PIPP and PIPP-r) [41, 42], the Neonatal Pain, Agitation and Sedation scale (N-PASS) [43], and continuous pain (the CRIES (acronym of Crying, Requires oxygen, Increased vital signs, Expression, Sleepless) scale [44], the Échelle Douleur Inconfort Nouveau-né (EDIN) scale [45], the Neonatal Pain, Agitation, and Sedation scale (N-PASS) [43], COMFORT-B [46], the COMFORTneo [47], and the Astrid Lindgren and Lund Children’s Hospital’s Pain and Stress Assessment scale for Preterm and Sick Newborn Infants (ALPS-Neo) [48]); retinopathy of prematurity; intraventricular hemorrhage; necrotizing enterocolitis; periventricular leukomalacia; bronchopulmonary dysplasia; constipation, and finally cerebral NIRS and aEEG. We will also include data on the duration of mechanical ventilation, oxygen supplementation, hospital stay, and the time to full enteral feeding. Furthermore, we will measure the frequency of major neurodevelopmental disabilities: cerebral palsy, developmental, intellectual impairment, blindness, or sensorineural deafness requiring amplification. For these, we plan to evaluate each of the components as a separate outcome and extract data on each long-term outcome from studies that evaluated children after 18 months’ chronological age, assessing data on children 18 to 24 months of age and on those 3 to 5 years of age separately. We will also include pharmacokinetic data (from observational studies): half-life, elimination time, and other reported pharmacokinetic measures.

### Search methods for identification of studies

We will conduct a systematic literature review search in the following databases: The Cochrane Central Register of Controlled Trials (CENTRAL, The Cochrane Library), MEDLINE (Ovid), and Embase and CINAHL for eligible studies to be included (Additional file 2). We will search https://clinicaltrials.gov and ICTRP for ongoing trials. No language and time restrictions will be applied.

### Searching other resources

Additionally, we will review the reference lists of all identified articles for any relevant articles that will not be identified in the primary search.

### Selection of studies

Two review authors will independently screen the titles and abstracts to identify potentially relevant citations, retrieve the full texts of all potentially relevant articles, and assess the eligibility of the studies. We will resolve any disagreements by discussion and, if necessary, by consulting a third review author. We will provide details of studies excluded from the review along with the reasons for exclusion. We will contact the trial authors if the details of the primary trials are unclear to request further information.

### Data extraction and management

Two review authors will independently undertake data abstraction using a data extraction form. We will extract the following characteristics from each included study:

- Administrative details: author(s), whether published or unpublished, year of publication, year in which study was conducted
- Details of study: study design, type, duration, and completeness of follow-up (e.g., greater than 80%), country and location of study informed consent, and ethics approval
- Details of participants: birth weight, gestational age, and number of participants
- Details of intervention: modality of administration and dose of the drugs
- Details of outcomes, as listed in types of outcome measures

We will resolve any disagreement by discussion between the review authors.

We will describe any ongoing studies identified, detailing the primary author, methods, and outcome measures.

If queries arise or when additional data are required, we will contact the authors of the trial reports. Two review authors will use the Review Manager 5 software (ReviewManager 2014, RevMan 5.4) to enter all the data [49].

### Assessment of risk of bias in included studies

Two review authors will independently assess the risk of bias (low, high, or unclear) of all included trials using the Cochrane “Risk of Bias” tool for the following domains [50]:

- Sequence generation (selection bias)
- Allocation concealment (selection bias)
- Blinding of participants and personnel (performance bias)
- Blinding of outcome assessment (detection bias)
- Incomplete outcome data (attrition bias)
- Selective reporting (reporting bias)
- Any other bias

We will resolve any disagreements by discussion or through a third review author.

For the included observational studies, we will use the “Risk Of Bias In Non-randomised Studies – of Interventions” (ROBINS-I) tool [51] to formally assess the risk of bias in the following domains: bias due to confounding, bias in selection of participants into the study, bias in classification of interventions, bias due to deviations from intended interventions, bias due to missing data, bias in measurement of outcomes, bias in the reported results, and the overall risk of bias. In the confounding domain, we took into account the following confounding factors: antenatal steroids, gestational age, birth weight, sex, Apgar score, antenatal exposure to opioids, indication to start opioids, and level of respiratory support at study entry.

### Measures of treatment effect

In general, we will extract categorical data for each intervention group and calculate risk ratios (RRs) and absolute risk differences (RDs). We will obtain means and standard deviations (SDs) for continuous data, and perform analyses using mean differences (MDs) when studies were measured in the same way across trials. We will use the standardized mean difference (SMD) to combine trials that measured the same outcome but used different methods. Where trials reported continuous outcomes as median and IQR and the data passed the test of skewness, we will convert mean to median and estimate the SD as IQR/1.35. If we find a variety of scales across studies, subgroup analysis will be performed pooling these measurements. For each measure of effect, we will calculate the corresponding 95% confidence intervals (CIs).

To specifically describe for the two primary outcomes, all-cause mortality during initial hospitalization will be assessed as RRs or RDs calculated from the numbers of infants who did or did not survive in each study arm. Hypotension requiring medical therapy (vasopressors, fluid boluses, or any changes in such drug administration) will also be assessed as RRs or RDs calculated from the number of infants who did or did not require such therapies in each study arm.

### Unit of analysis issues

The unit of analysis will be the individual infant. For multiple painful procedures, we will consider the first procedure performed in the randomized infant.

### Dealing with missing data

We will contact the original study investigators to request additional data where information about critical and important outcomes will be missing. We will investigate attrition rates (e.g., dropouts, losses to follow-up, and withdrawals). We will perform a sensitivity analysis to evaluate the overall results with and without the inclusion of studies with significant dropout rates. If a study will report outcomes only for participants completing the trial, or only for participants who followed the protocol, we will contact the authors and ask them to provide additional information to facilitate an intention-to-treat analysis. We will address the potential impact of missing data on the findings of the review in the “Discussion” section of the final manuscript.

### Assessment of heterogeneity

We will assess clinical heterogeneity by comparing the distribution of important participant factors between trials and trial factors (e.g., randomization concealment, blinding of outcome assessment, loss to follow-up, treatment type, co-interventions). We will assess statistical heterogeneity by examining the *I*^2^ statistic [50], a quantity that describes the proportion of variation in point estimates that is due to variability across studies rather than sampling error.

We will interpret the *I*^2^ statistic as described by Higgins 2003 [52]:

- Less than 25%: no heterogeneity
- 25% to 49%: low heterogeneity
- 50% to 74%: moderate heterogeneity
- 75% or greater: high heterogeneity

In case of high heterogeneity (*I*^2^ equal to or greater than 75%), we will not pool the studies in the meta-analysis. In addition, we will employ the chi^2^ test of homogeneity to determine the strength of evidence that heterogeneity is genuine. We will explore clinical variation across studies by comparing the distribution of important participant factors among trials and trial factors (randomization concealment, blinding of outcome assessment, loss to follow-up, treatment type, and co-interventions). We will consider a threshold *P* value of less than 0.1 as an indicator of whether heterogeneity (genuine variation in effect sizes) is present.

### Assessment of reporting biases

We will create and examine a funnel plot to explore possible small-study biases. In interpreting funnel plots, we will examine the different possible reasons for funnel plot asymmetry as outlined in section 10.4 of the Handbook and relate this to the results of the review. If we are able to pool more than 10 trials, we will undertake formal statistical tests to investigate funnel plot asymmetry, and will follow the recommendations in section 10.4 of the Handbook [50, 53].

### Data synthesis

We will perform statistical analyses according to the recommendations of the Cochrane Neonatal Review Group (neonatal.cochrane.org/en/index.html). We will analyze all infants randomized on an intention-to-treat basis. For any meta-analyses, we will synthesize data using RR, RD, number needed to treat to benefit (NNTB), number needed to treat to harm (NNTH), MD, and 95% CI. For our two primary outcomes, we will assess whether the mean and 95% CI of overall RRs are below or above the null of 1. We will analyze and interpret individual trials separately when we judged meta-analysis to be inappropriate.

### Subgroup analysis and investigation of heterogeneity

If enough RCTs will be included, we will perform a subgroup analysis for the following:

- Gestational age
- Postnatal age
- Indication (e.g., sedation vs analgesia)
- Type of pain (e.g., procedural, postoperative, or other painful condition, sedation for mechanical ventilation)
- Type of administration (loading dose or not, bolus vs continuous infusion)
- Route of administration (enteral vs intravenous, between other routes)
- Specific conditions affecting pharmacokinetics (e.g., on extracorporeal membrane oxygenation (ECMO) or not, on hypothermia or not)
- Combination of administration (effect of target drug compared to a specific drug or combination of drugs)

### Sensitivity analysis

We will conduct sensitivity analyses to explore the effect of the methodological quality of the trials, checking to ascertain if studies with a high risk of bias overestimated the effect of treatment.

### Summary of findings and assessment of the certainty of the evidence

We planned to use the GRADE approach, as outlined in the GRADE Handbook to assess the quality of evidence for the following clinically relevant outcomes (gdt.guidelinedevelopment.org/central_prod/_design/client/handbook/handbook.html) [54]:

1. All-cause neonatal mortality (death until postnatal day 28)
2. All-cause mortality during initial hospitalization
3. Hypotension requiring medical therapy (vasopressors, fluid boluses, or any changes in such drug administration)
4. Episodes of bradycardia defined as a fall in heart rate of more than 30% below the baseline or less than 100 beats per minute for 10 s or longer
5. Respiratory depression, i.e., episodes of apnea (mean rates of apnea)
6. Pain during the administration of selected drugs
7. Sedation during the administration of selected drugs

Two review authors will independently assess the certainty of the evidence for each of the outcomes above. We will consider evidence from RCTs as high certainty but downgrade the evidence one level for serious (or two levels for very serious) limitations based upon the following: design (risk of bias), consistency across studies, directness of the evidence, precision of estimates, and presence of publication bias. We will use the GRADEpro GDT software to create a “Summary of findings” table to report the certainty of the evidence.

The GRADE approach results in an assessment of the certainty of a body of evidence as being one of the following four grades:

- High: we are very confident that the true effect lies close to that of the estimate of the effect
- Moderate: we are moderately confident in the effect estimate; the true effect is likely to be close to the estimate of the effect, but there is a possibility that it is substantially different
- Low: our confidence in the effect estimate is limited; the true effect may be substantially different from the estimate of the effect
- Very low: we have very little confidence in the effect estimate; the true effect is likely to be substantially different from the estimate of effect

## Discussion

Since the late 1990s, there has been a huge interest in the domain of pain-related issues in the newborn population. A large amount of studies with different study designs and varying quality has been conducted in different areas including microbiology, pharmacology, neurophysiology, and caring strategies. “The EU Regulation on medical products for paediatric use” came into use in 2007, stating that all drugs used should have been studied on the specific patient population to which they are administered [55], and this started an era of intensive research in neonatal pharmacology. Since then, the pharmacokinetic modeling [56] has allowed to expand the research opportunities in neonatal medicine, e.g., the enhanced possibilities for pharmacokinetic/pharmacodynamic (PK/PD) studies.

However, the situation varies in the NICU; the infants differ in regard to gestational and postnatal age, illness, diagnoses, and most of all the type of clinical care varies between hospitals and countries. The need for evidence-based national and international guidelines is of utmost importance. The optimal platform for developing safe recommendations and such guidelines would be initiated by a systematic review.

## Supplementary information

**Additional file 1:.** PRISMA-P checklist

**Additional file 2:.** Search strategy used for online databases

## Data Availability

Not applicable.

## Abbreviations

NICU: Neonatal intensive care unit
IQR: Interquartile range
PK: Pharmacokinetics
PD: Pharmacodynamics
EEG: Electroencephalography
NIRS: Near-infrared spectroscopy
RCT: Randomized controlled trial
PMA: Postmenstrual age
aEEG: Amplitude integrated electroencephalography
RR: Risk ratio
RD: Risk difference
SD: Standard deviation
MD: Mean difference
SMD: Standardized mean difference
CI: Confidence interval
NNTB: Number needed to treat to benefit
NNTH: Number needed to treat to harm
