# Supplementary material for: Opioids and alpha-2-agonists for analgesia and sedation in newborn infants: protocol of a systematic review
Source: Syst Rev. 2020 Aug 20;9:183. doi: 10.1186/s13643-020-01436-0 (PMC7441710; doi:10.1186/s13643-020-01436-0)
Supplement: Supplementary file 2 — Additional file 2:. Search strategy used for online databases [file 13643_2020_1436_MOESM2_ESM.docx]

**Databases:**

(((infant, newborn[MeSH] OR newborn*[TIAB] OR “new born”[TIAB] OR “new borns”[TIAB] OR “newly born”[TIAB] OR

baby*[TIAB] OR babies*[TIAB] OR premature[TIAB] OR prematurity[TIAB] OR preterm[TIAB] OR “pre term”[TIAB] OR “low

birth weight”[TIAB] OR “low birthweight”[TIAB] OR VLBW[TIAB] OR LBW[TIAB] OR infan*[TIAB] OR neonat*[TIAB])))

AND

((((((morphine OR diamorphine OR fentanyl OR alfentanil OR sufentanil OR pethidine OR meperidine OR codeine

OR methadone))) OR (“Narcotics”[Majr] OR “Analgesia”[Majr] OR sedation[Title/Abstract] OR opioid*[Title/Abstract]

OR remifentanil)) OR ((((((((“Morphine”[Mesh]) OR “Heroin”[Mesh]) OR “Fentanyl”[Mesh]) OR “Alfentanil”[Mesh]) OR

“Sufentanil”[Mesh]) OR “Meperidine”[Mesh]) OR “Codeine”[Mesh]) OR “Methadone”[Mesh] OR “Remifentanil”[Mesh]))

OR

((Alpha-2 agonists[Title/Abstract] OR clonidine[Title/Abstract]) OR Clonidine[Mesh] OR Adrenergic alpha-2 Receptor Agonists [Pharmacological Action] OR alpha 2 agonists, adrenergic[Mesh])

OR

(Dexmedetomidine[Mesh] OR Dexmedetomidine[Title/Abstract]))

AND

(randomized controlled trial[pt] OR controlled clinical trial[pt] OR randomized[tiab] OR randomised[tiab] OR randomly[tiab] OR placebo[tiab] OR drug therapy[sh] OR randomly[tiab] OR trial[tiab] OR groups[tiab]) NOT (animals[MH] NOT humans[MH]))

OR

(Clinical trial[Title/Abstract] OR observational[Title/Abstract] OR case-control[Title/Abstract] OR “case control”[Title/Abstract] OR retrospective[Title/abstract] OR “multi-center study”[Title/Abstract] OR “multi center study”[Title/Abstract] OR “multi-centre study”[Title/Abstract] OR “multi centre study”[Title/Abstract])

No language restriction or publication date restriction was used.

## Embase (Elsevier)

’prematurity’/exp OR ’infant’/exp OR newborn*:ti,ab OR ’new born’:ti,ab OR ’new borns’:ti,ab OR ’newly born’:ti,ab OR baby*:ti,ab OR babies:ti,ab OR premature:ti,ab OR prematurity:ti,ab OR preterm:ti,ab OR ’pre term’:ti,ab OR ’low birth weight’:ti,ab OR ’low birthweight’:ti,ab OR vlbw:ti,ab OR lbw:ti,ab OR infant:ti,ab OR infants:ti,ab OR infantile:ti,ab OR infancy:ti,ab OR neonat*:ti,ab

AND

narcotic AND analgesic AND 'agent'/exp/mj OR 'analgesia'/exp/mj OR sedation:ti,ab OR morphine:ab,ti OR 'diamorphine':ab,ti OR 'fentanyl':ab,ti OR 'alfenatil':ab,ti OR 'sufentanil':ab,ti OR 'pethidine':ab,ti OR 'meperidine':ab,ti OR 'codeine':ab,ti OR 'methadone':ab,ti OR 'morphine'/exp OR 'diamorphine'/exp OR 'fentanyl'/exp OR 'alfentanil'/exp OR 'sufentanil'/exp OR 'pethidine'/exp OR 'methadone'/exp OR 'remifentanil':ab,ti OR 'remifentanil'/exp OR opioid*:ti,ab OR 'clonidine'/exp OR 'alpha 2 adrenergic receptor stimulating agent'/exp OR 'alpha 2 agonist*':ab,ti OR clonidine:ab,ti OR 'dexmedetomidine'/exp OR dexmedetomidine:ab,ti

AND

'randomized controlled trial'/exp OR 'randomized controlled trial' OR 'controlled clinical trial'/exp OR 'controlled clinical trial' OR 'randomized':ab,ti OR 'randomised':ab,ti OR 'placebo':ab,ti OR 'randomly':ab,ti OR 'trial':ab,ti OR 'clinical trial'/exp OR 'clinical trial' OR observational:ti,ab OR 'case-control':ti,ab OR 'case control':ti,ab OR retrospective:ti,ab OR 'multi-center study':ti,ab OR 'multi center study':ti,ab OR 'multi-centre study':ti,ab OR 'multi centre study':ti,ab

NOT ‘conference abstract’:it

No language restriction or publication date restriction was used.

## Cochrane CENTRAL

#1 MeSH Descriptor: [Infant, newborn] EXPLODE ALL

#2 (infan* or newborn* or “new born” or “new borns” or “newly born” or neonat* or baby* or babies or premature or prematures or prematurity or preterm* or “pre term” or premies or “low birth weight” or “low birthweight” or VLBWor LBW or ELBW or NICU):ti,ab,kw

#3 (morphine OR diamorphine OR fentanyl OR alfentanil OR sufentanil OR pethidine OR meperidine OR codeine OR methadone OR remifentanil OR clonidine OR alpha-2 agonists OR Adrenergic alpha-2 Receptor Agonists OR Dexmedetomidine:ti,ab,kw

#4 (#1 OR #2) AND #3

## CINAHL Complete (Ebsco)

(infant or infants or infantile or infancy or newborn* or “new born” or “new borns” or “newly born” or neonat* or baby* or babies or premature or prematures or prematurity or preterm or preterms or “pre term” or premies or “low birth weight” or “low birthweight” or VLBW or LBW)

AND

(morphine OR diamorphine OR fentanyl OR alfentanil OR sufentanil OR pethidine OR meperidine OR codeine OR methadone

OR MH morphine OR MH diamorphine OR MH fentanyl OR MH alfentanil OR MH sufentanil OR MH pethidine OR MH

meperidine OR MH codeine OR MH methadone OR MH remifentanil OR MJ narcotics OR MJ sedation OR MJ analgesia OR TI OR opioid* OR AB opioid*)

OR MH clonidine OR TI clonidine OR AB clonidine OR
MH Adrenergic Alpha-Agonists OR TI Adrenergic Alpha-Agonists OR AB Adrenergic Alpha-Agonists OR TI alpha 2 agonist OR AB alpha 2 agonist OR MH Dexmedetomidine OR TI Dexmedetomidine OR AB Dexmedetomidine

AND

(TX “randomized controlled trial” OR TX “controlled trial” OR TX randomized OR TX placebo OR TX “clinical trials as topic”

OR TX randomly OR TX trial OR PT clinical trial) OR TI (Clinical trial OR observational OR case-control OR “case control” OR retrospective OR “multi-center study” OR “multi center study” OR “multi-centre study” OR “multi centre study ) OR AB ( Clinical trial OR observational OR case-control OR “case control” OR retrospective OR “multi-center study” OR “multi center study” OR “multi-centre study” OR “multi centre study)

**Ongoing studies:**

## Clinicaltrials.gov

Search field: Other terms

(Infant OR newborn OR premature)

AND

(sedation OR analgesia OR opioids OR clonidine OR Dexmedetomidine OR alpha 2 agonists)

## ICTRP 200116

(infant OR newborn OR premature) AND (sedation OR analgesia OR opioids OR clonidine OR Dexmedetomidine OR alpha 2 agonists) in Title field
